# The trend of poisonings before and after the COVID-19 pandemic

**DOI:** 10.1038/s41598-024-52537-y

**Published:** 2024-01-24

**Authors:** Amir Hossein Behnoush, Elham Bazmi, Amirmohammad Khalaji, Amirhossein Jafari-Mehdiabad, Nasrin Barzegari, Ahmad-Reza Dehpour, Behnam Behnoush

**Affiliations:** 1 School of Medicine, Tehran University of Medical Sciences, Tehran, Iran; 2 Legal Medicine Research Center, Legal Medicine Organization, Tehran, Iran; 3 Department of Psychiatry, Roozbeh Hospital, Tehran University of Medical Sciences, Tehran, Iran; 4 School of Medicine, Baharloo Hospital, Tehran University of Medical Sciences, Tehran, Iran; 5 Experimental Medicine Research Center, Tehran University of Medical Sciences, Tehran, Iran; 6 Department of Pharmacology, School of Medicine, Tehran University of Medical Sciences, Tehran, Iran; 7 Department of Forensic Medicine, Tehran University of Medical Sciences, Tehran, Iran

**Keywords:** Viral infection, Epidemiology, Health policy

## Abstract

The COVID-19 pandemic has substantially affected people and healthcare systems. One of the main challenges was the reduction and change in the pattern of non-COVID-19 diseases and conditions. Moreover, due to the mental burden of the pandemic, the trend of poisonings and abuses changed. In this study, we aimed to assess the trends of poisonings from different agents before and during the COVID-19 pandemic using the interrupted time series method. This study was conducted at one of the main Tehran referral centers for poisoning, Baharloo Hospital. Pre-COVID-19 period was defined as April 2018 to January 2020 while the COVID-19 time was from February 2020 to March 2022. The total number of monthly poisoning cases in addition to eight categories of drugs/substances/agents were identified, including drugs (such as psychiatric drugs, cardiovascular drugs, and analgesics), opioids, stimulants, methanol, ethanol, cannabis, pesticides, and carbon monoxide. Interrupted time series analysis was performed to compare the pre-pandemic trend of total monthly cases from each category in addition to the proportion (%) of each one. In total, 13,020 cases were poisoned during the study period, among which 6088 belonged to the pre-pandemic period and 6932 were admitted during the COVID-19 era. There was no significant difference in terms of demographic characteristics of patients before and during the pandemic (p-value > 0.05). At the beginning of the pandemic, there was a sudden fall in the number of poisoning patients (− 77.2 cases/month, p-value = 0.003), however, there was a significant increasing trend during the COVID time (3.9 cases/month, p-value = 0.006). Most of the categories had a sharp decrease at the beginning of the pandemic except for methanol and ethanol which had increases, although not significant. Cannabis also had a significant change in slope (− 0.6 cases/month, p-value = 0.016), in addition to the sudden decrease at the beginning of the pandemic (− 10 cases/month, p-value = 0.007). Regarding the proportion of each category from total monthly poisoning cases, methanol, and ethanol had immediate rises of 4.2% per month and 10.1% per month, respectively (both significant). The pandemic had significant effects on the pattern of poisonings from different agents in Iran, the most important of which were alcohol (ethanol and methanol). These differences had policy implications that can be helpful for policymakers and healthcare systems in combating similar situations in the future.

## Introduction

The pandemic caused by severe acute respiratory syndrome coronavirus 2 (SARS‑CoV‑2) changed the patterns of hospitalizations in several medical conditions^1^. Although the coronavirus disease of 2019 (COVID-19) led to the hospitalization of a large number of patients worldwide^2^, a decrease was observed in hospitalizations due to other medical conditions requiring acute care^1^; which was reported to be up to 32% in a study^3^. They found the most significant decreases in asthma, chronic obstructive pulmonary disease (COPD), and heart failure admissions. However, respiratory-related admissions (e.g., respiratory failure) and hospitalization following traumatic brain injury increased. Moreover, the pandemic affected hospital admissions and readmissions related to the mental health^4^ which could lead to higher drug and substance overdose.

The COVID-19 pandemic has also changed the trend of hospitalizations related to poisoning as well as deaths related to poisonings^5,6^. For instance, Hadeiy et al. found a higher rate of alcohol intoxication and intoxication-related mortality during the COVID-19 pandemic compared to before^7^. Moreover, opioid overdose was reported to be higher than pre-COVID-19 than during the pandemic in the United States^8^. Another retrospective study found a lower rate of patients with carbon monoxide (CO) intoxication needing treatment in Germany^9^. Using interrupted time series analysis, a nationwide study among young children in the United States showed that there was an immediate increase in the ingestion of cannabis, opioids, and ethanol^10^.

Several studies compared hospitalizations due to alcohol intoxications before and during the COVID-19 pandemic^7,11,12^, while some also investigated cannabis use in some limited populations^13^. However, there is not much evidence regarding hospitalization trends of these two types of poisoning in large-scale studies and also there is no literature regarding the poisoning with other substances such as opioids, CO gas, stimulants, and pesticides before and during the pandemic. Therefore, comparing the trends and slope of hospitalizations before and during COVID-19, as well as the sudden change at the start of the pandemic, can help policymakers update guidelines for informing, preventing, and treating poisonings, and also may be useful in preparation for following pandemics. In this study, we used interrupted time series analysis to compare hospitalizations due to poisonings in Tehran, Iran before and during COVID-19. We investigated the number of each poisoning and the ratio of them before and after the COVID-19 pandemic with a monthly interrupted time series method. This method has been used in several similar instances and for COVID-19^7,14,15^.

## Methods

### Study population and design

This retrospective cross-sectional study was performed in Baharloo Hospital, a referral hospital for poisoning and COVID-19 patients in Tehran, Iran. Hospitalized patients from April 2018 to March 2022 who were confirmed poisoning cases entered the study. The Iranian Ministry of Health announced the official initiation of the COVID-19 pandemic in Iran in February 2020. Therefore, the pre-COVID-19 and post-COVID pandemics were defined as April 2018–January 2020 and February 2020 to March 2022, respectively. The following baseline characteristics were collected from electronic health records (EHR) and clinical documents: sex, age, marital status, job status, residency, physiological disorders, addiction history, suicide history, and cause of poisoning were compared between pre- and post-COVID-19 periods.

### Types of drugs/poisonings

The following nine groups of substances causing poisoning were detected from the manifestation of patients and toxicological analysis (screen tests and confirmation tests).

(1) Ethanol intoxication (ICD-10 code: T51.0, F10.12); (2) Methanol intoxication, (ICD-10 codes: T51.1); (3) Opioid intoxication, which includes natural or synthetic opioids and opioid drugs such as tramadol, methadone drugs, detected (ICD-10 codes: T40.0, T40.1, T40.2, T40.3, T40.4, and T40.6); (4) Stimulant intoxication include drugs such as cocaine and amphetamines, (ICD-10 codes: T43.6, and T40.5); (5) Drug intoxication, defined as anti-psychotics, anti-depressants, anti-convulsants, cardiovascular drugs, and analgesics (ICD-10 codes: T43.0, T43.1, T43.2, T43.4, T43.4, T43.5, and T46); (6) CO poisoning (ICD-10: T58), (7) Cannabis intoxication (ICD-10: T40.7, F12.12), and (8) Pesticide intoxication (ICD-10: T60).

### Statistical analysis

In descriptive and analytical analysis, data were presented as mean ± standard deviation (SD) for continuous variables and number (percentage) for categorical variables. Chi-squared test and independent t-test were utilized for evaluating differences between pre- and post-COVID-19 periods, for categorical and continuous variables, respectively. The Kolmogorov–Smirnov test was used to evaluate the normal distribution of data.

Interrupted time series analysis (ITS) is a quasi-experimental time series analysis that involves statistical analysis of tracking a long-term period before and after a point of intervention such as the COVID-19 epidemic. In this method, outcomes are measured constantly at different time points before and after special events and the change in trend and levels of those particular outcomes are determined. The Newey–West method was used to conduct ITS incorporating autocorrelation assumption by defining a lag and based on the Breusch–Godfrey (BG) approach^16–18^. ITS was used for totally poisoned patients and each group of substances that caused poisoning. Also, the proportion of each type of substance was separately analyzed. Finally, the test of Dickey and Fuller was used to assess the non-stationary assumption^19^.

STATA 17 (Stata Corp LLC, Texas, USA) was used to perform ITS and measurement of trends during the pre- and post-COVID-19 period. A p-value of ≤ 0.05 was considered statistically significant.

### Ethics approval and consent to participate

All participants provided written informed consent. This study was approved by the ethics committee of the Tehran University of Medical Sciences (IR.TUMS.MEDICINE.REC.1400.1330) and was conducted in accordance with the Declaration of Helsinki.

## Results

### Baseline characteristics of patients

A total of 13,020 patients were poisoned from April 2018 to March 2022, of which 6088 were during the pre-COVID time and 6932 during the COVID-19 pandemic. The mean age of patients was 32.42 ± 9.11 years (mean ± SD) and 8007 (61.5%) were male. Most of the patients were single (68.7%) and 90% lived in urban areas. The rate of unemployment increased from 18% in the pre-pandemic era to 20.2% during the pandemic, which was not statistically significant (p-value = 0.06). Table 1 shows all baseline characteristics of all patients and also before and after COVID-19. There was no statistical difference between sex, age, marital status, job status, residence area, psychological disorder, addiction history, and suicide history before and after COVID-19.

**Table 1:** Demographic characteristics of poison before and during the COVID-19 pandemic.

Demographic characteristics	All poisonings Apr 2018 to Mar 2022 (N = 13,020) N (%)	Before COVID-19 Apr 2018 to Jan 2020 (N = 6088) N (%)	During COVID-19 Feb 2020 to Mar 2022 (N = 6932) N (%)	p-value
Sex				
Male	8007 (61.5)	3799 (62.4)		0.16
Female	5013 (38.5)	2289 (37.6)	2724 (39.3)	
Age (mean ± SD) years	32.42 ± 9.11	33.6 ± 9.41	31.24 ± 8.31	0.16
Marital status				
Single	8948 (68.7)	4304 (70.7)	4644 (67)	0.22
Married	3866 (29.7)	1662 (27.3)	2204 (31.8)	
Unknown	206 (1.6)	122 (2)	84 (1.2)	
Job status				
Unemployed	2496 (19.2)	1096 (18)	1400 (20.2)	0.06
Housewife	3059 (23.5)	1278 (21)	1781 (25.7)	
Government	342 (2.6)	231 (3.8)	111 (1.6)	
Private	2762 (21.2)	1254 (20.6)	1511 (21.8)	
Students	1239 (9.5)	560 (9.2)	679 (9.8)	
Unknown	3119 (23.9)	1669 (27.4)	1450 (20.9)	
Residency				
Urban	11,726 (90)	5418 (89)	6308 (91)	0.84
Rural	1294 (10)	670 (11)	624 (9)	
Psychological disorders				
Yes	930 (7.1)	597 (9.8)	333 (4.8)	0.07
No	12,090 (92.9)	5491 (90.2)	6599 (95.2)	
Addiction history				
Yes	3238 (24.9)	1644 (27)	1594 (23)	0.67
No	9782 (75.1)	4444 (73)	5338 (77)	
Suicide history				
Yes	1680 (12.9)	779 (12.8)	901 (13)	0.45
No	11,340 (87.1)	5309 (87.2)	6031 (87)	

Data are presented as numbers (percentage) or mean (standard deviation).

### Total poisoning trend

**Figure 1:**
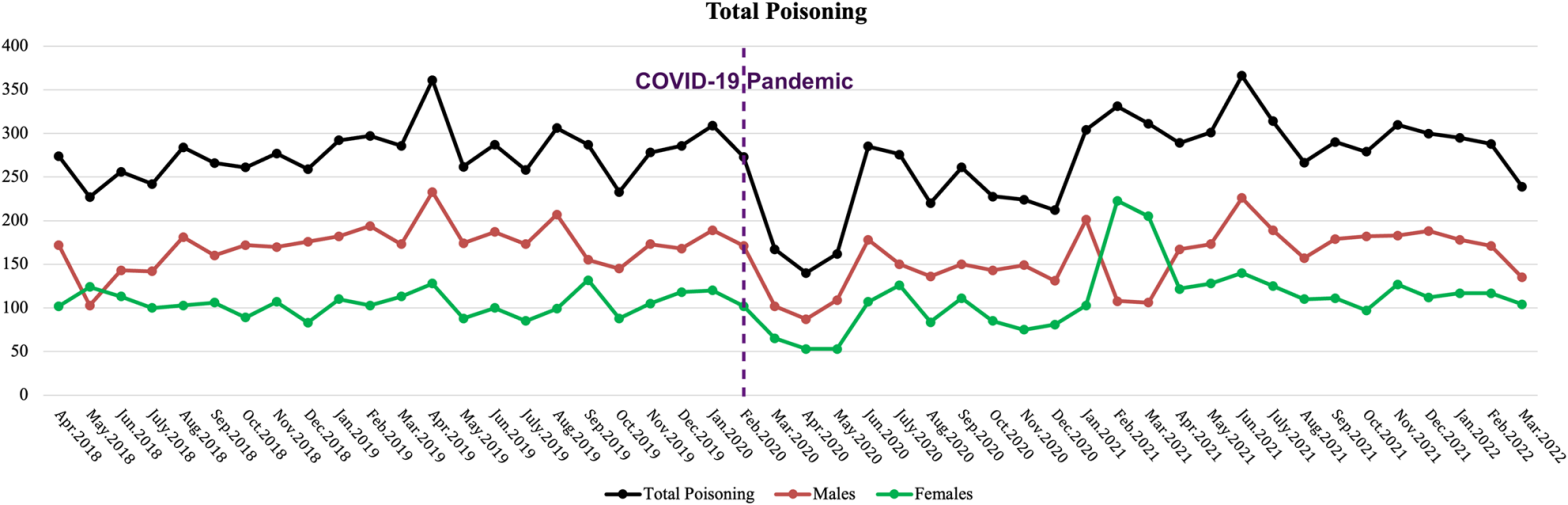
Total poisoning trend in the population before and after the COVID-19 pandemic for males, females, and both sexes.

The poisoning trend was ascending before COVID-19 initiation with a significant increase of 1.6 monthly cases (95% CI 0.023 to 3.171, p-value = 0.047). At the start of the COVID-19 pandemic, there was a sudden significant decrease in poisonings (− 77.211 cases/month, 95% CI − 126.662 to − 27.759, p-value = 0.003). However, the change in the slope of the trend was not significant (p-value = 0.148). Also, the trend slope was significant in an increasing manner during the COVID-19 pandemic (p-value = 0.006). All of these are described in Table 2 and Fig. 1.

**Table 2:** The interrupted time series analysis for the total poisoning number per month.

Variable	Coefficient	Standard error	p-value	95% CI
Constant	258.364	9.265	< 0.001	239.69 to 277.037
Slope before	1.597	0.781	**0.047**	0.023 to 3.171
Level change	− 77.211	24.537	**0.003**	− 126.662 to − 27.759
Slope change	2.302	1.561	0.148	09.845 to 5.449
Slope after	3.899	1.352	**0.006**	1.174 to 6.624

Significant values are in bold.

### Interrupted time series analysis of poisoning numbers by each substance/drug

#### Drugs

**Figure 2:**
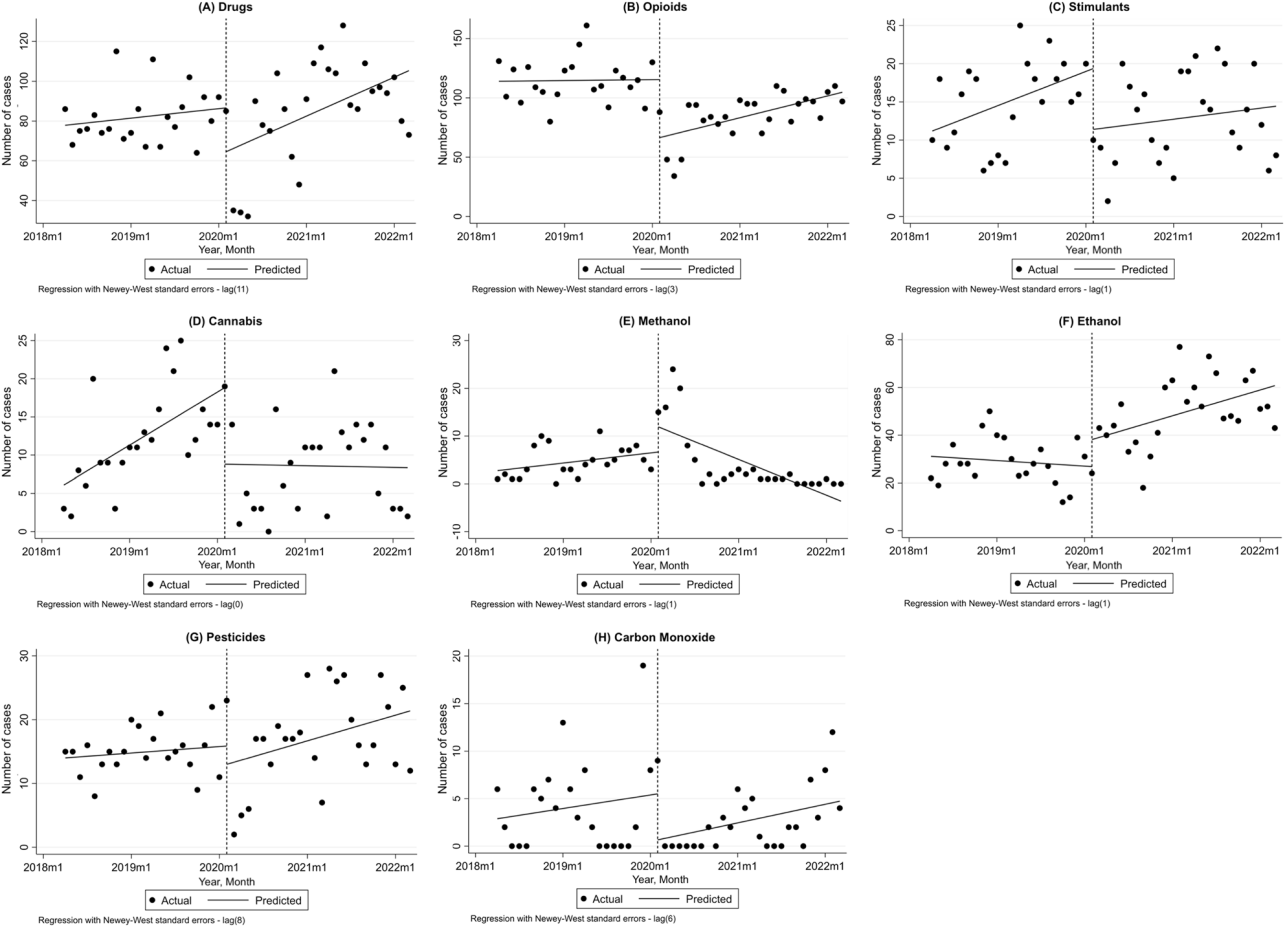
Interrupted time series analysis of the absolute number of poisonings from each category before and after the COVID-19 pandemic; (**A**) Drugs, (**B**) Opioids, (**C**) Stimulants, (**D**) Cannabis, (**E**) Methanol, (**F**) Ethanol, (**G**) Pesticides and (**H**) Carbon Monoxide; Dashed line represents COVID-19 outbreak; *m* month.

Figure 2A represents the trend for drug poisonings and the ratio of drug poisonings before and after COVID-19. In total, 4013 drug poisonings were recorded from April 2018 to March 2022, among which 1805 were before February 2020 and 2208 were reported during the pandemic. As Table 3 shows, before the pandemic drug poisoning was at a significantly increasing trend of 0.4 cases per month while at the initiation of the COVID-19 pandemic in Iran, a significant drop of − 22.08 monthly cases was observed (p-value = 0.021).

**Table 3:** The interrupted time series analysis for absolute poisoning numbers due to drugs, opioids, stimulants, and cannabis.

Variable	Coefficient	Standard error	p-value	95% CI
Drugs				
Constant	77.481	1.579	< 0.001	74.299 to 80.662
Slope before	0.397	0.114	**0.001**	− 40.598 to − 3.562
Level change	− 22.08	9.188	**0.021**	− 40.598 to − 3.562
Slope change	1.234	0.655	0.066	− 0.086 to 2.556
Slope after	1.631	0.648	**0.016**	0.324 to 2.938
Opioids				
Constant	113.987	6.066	< 0.001	101.761 to 126.213
Slope before	0.064	0.352	0.856	− 0.645 to 0.774
Level change	− 49.04	9.741	** < 0.001**	− 68.671 to − 29.41
Slope change	1.467	0.508	**0.006**	0.443 to 2.492
Slope after	1.532	0.408	** < 0.001**	0.71 to 2.354
Stimulants				
Constant	10.818	2.123	< 0.001	6.537 to 15.098
Slope before	0.371	0.119	**0.003**	0.131 to 0.612
Level change	− 7.962	2.985	**0.011**	− 13.978 to − 1.945
Slope change	− 0.249	0.215	0.252	− 0.684 to 0.184
Slope after	0.122	0.18	0.502	− 0.241 to 0.484
Cannabis				
Constant	5.519	2.128	0.013	1.23 to 9.809
Slope before	0.579	0.168	**0.001**	0.240 to 0.918
Level change	− 10.041	3.521	**0.007**	− 17.137 to − 2.944
Slope change	− 0.597	0.239	**0.016**	− 1.079 to − 0.116
Slope after	− 0.018	0.17	0.916	− 0.36 to 0.324

Significant values are in bold.

#### Opioids

There were 2524 opioid poisoning before the pandemic and 2225 cases during the pandemic, the trend of which is illustrated in Fig. 2B. Before the pandemic, there was a slight insignificant increasing trend in opioid poisonings that changed significantly after the pandemic (1.47 cases per month, p-value = 0.006, Table 3) and led to an increasing trend of 1.53 monthly cases during the pandemic (p-value < 0.001). Moreover, at the beginning of the pandemic, a sudden decrease of − 49.04 monthly cases of opioid poisoning was observed (p-value = 0.006).

#### Stimulants

Before the pandemic, there were 332 patients with stimulant poisoning, and 336 cases during the COVID-19 pandemic course. As shown in Fig. 2C and Table 3, before the pandemic, stimulants had an increasing trend of 0.371 monthly cases (95% CI 0.131 to 0.612, p-value = 0.003). At the time of the COVID-19 pandemic initiation in Iran, a decrease of 8 cases per month was observed (95% CI − 13.98 to − 1.94, p-value = 0.011). However, the changes in slope and slope after COVID-19 were not significant.

### Cannabis

A total of 491 patients were admitted due to poisoning, among which 268 were before the pandemic. There was an increasing trend in cannabis poisoning before February 2020 (0.579 cases/month, 95% CI 0.240 to 0.918, p-value = 0.001, Table 3, Fig. 2D). While there was a sudden decrease in February 2020 (p-value = 0.007), the slope decreased significantly (− 0.597, 95% CI − 1.079 to − 0.116, p-value = 0.016).

#### Methanol

Regarding methanol poisoning, there were 101 methanol poisoning patients before February 2020 and 108 during the pandemic. As shown in Fig. 2E and Table 4, there was an insignificant increasing trend of 0.18 cases per month which had a sudden increase of 5.3 monthly cases at the time of COVID-19 initiation, although insignificant. The trend slope changed in a decreasing manner (− 0.798, 95% CI − 1.233 to − 0.363, p-value = 0.001), resulting in a decreasing trend of − 0.622 cases (p-value = 0.003).

**Table 4:** The interrupted time series analysis for absolute poisoning numbers due to methanol, ethanol, pesticides, and CO.

Variable	Coefficient	Standard error	p-value	95% CI
Methanol				
Constant	2.558	1.563	0.109	− 0.592 to 5.708
Slope before	0.177	0.098	0.081	− 0.022 to 0.376
Level change	5.3	3.709	0.160	− 2.176 to 12.776
Slope change	− 0.798	0.216	**0.001**	− 1.233 to − 0.363
Slope after	− 0.622	0.195	**0.003**	− 1.014 to − 0.229
Ethanol				
Constant	31.299	4.976	< 0.001	21.271 to 41.327
Slope before	− 0.196	0.374	0.604	− 0.951 to 0.559
Level change	11.370	7.267	0.125	− 3.275 to 26.016
Slope change	1.1	0.49	**0.030**	0.113 to 2.087
Slope after	0.904	0.325	**0.008**	0.249 to 1.559
Pesticides				
Constant	13.935	1.066	< 0.001	11.788 to 16.082
Slope before	0.085	0.079	0.292	− 0.075 to 0.245
Level change	− 2.883	2.336	0.224	− 7.59 to 1.824
Slope change	0.251	0.177	0.164	− 0.106 to 0.607
Slope after	0.335	0.148	**0.029**	0.036 to 0.634
CO				
Constant	2.766	1.597	0.090	− 0.453 to 5.985
Slope before	0.119	0.164	0.472	− 0.212 to 0.450
Level change	− 4.848	2.535	0.062	− 9.956 to 0.259
Slope change	0.043	0.217	0.841	− 0.393 to 0.480
Slope after	0.163	0.103	0.122	− 0.045 to 0.371

Significant values are in bold.

#### Ethanol

A total of 639 and 1286 patients had ethanol poisoning before and after the COVID-19 era, respectively. As shown in Fig. 2F, the slope of the ethanol poisoning trend changed from diminishing to rising after the COVID-19 pandemic in Iran. This change was significant (1.1, 95% CI 0.113 to 2.087, p-value = 0.030, Table 4). A monthly increase of 0.904 cases was observed since February 2020 (p-value = 0.008).

#### Pesticides

Totally 775 patients were admitted due to pesticide poisoning and 328 (42.3%) were before the pandemic. There was no significant trend before COVID-19 in terms of pesticide poisoning (p-value = 0.292, Table 4). Also, the change in slope was increasing despite being insignificant which resulted in a significant trend of 0.335 cases/month during the COVID-19 period (p-value = 0.029) (Fig. 2G).

#### CO

Before the pandemic, 91 patients presented with CO poisoning while 70 patients were after COVID-19 initiation in the country. CO poisoning had no significant trend before and after COVID-19 (p-value = 0.472 and 0.122, respectively) (Fig. 2H). As described in Table 4, the level change and slope change were not significant as well.

### Interrupted time series analysis of poisoning ratio by each substance/drug

**Figure 3:**
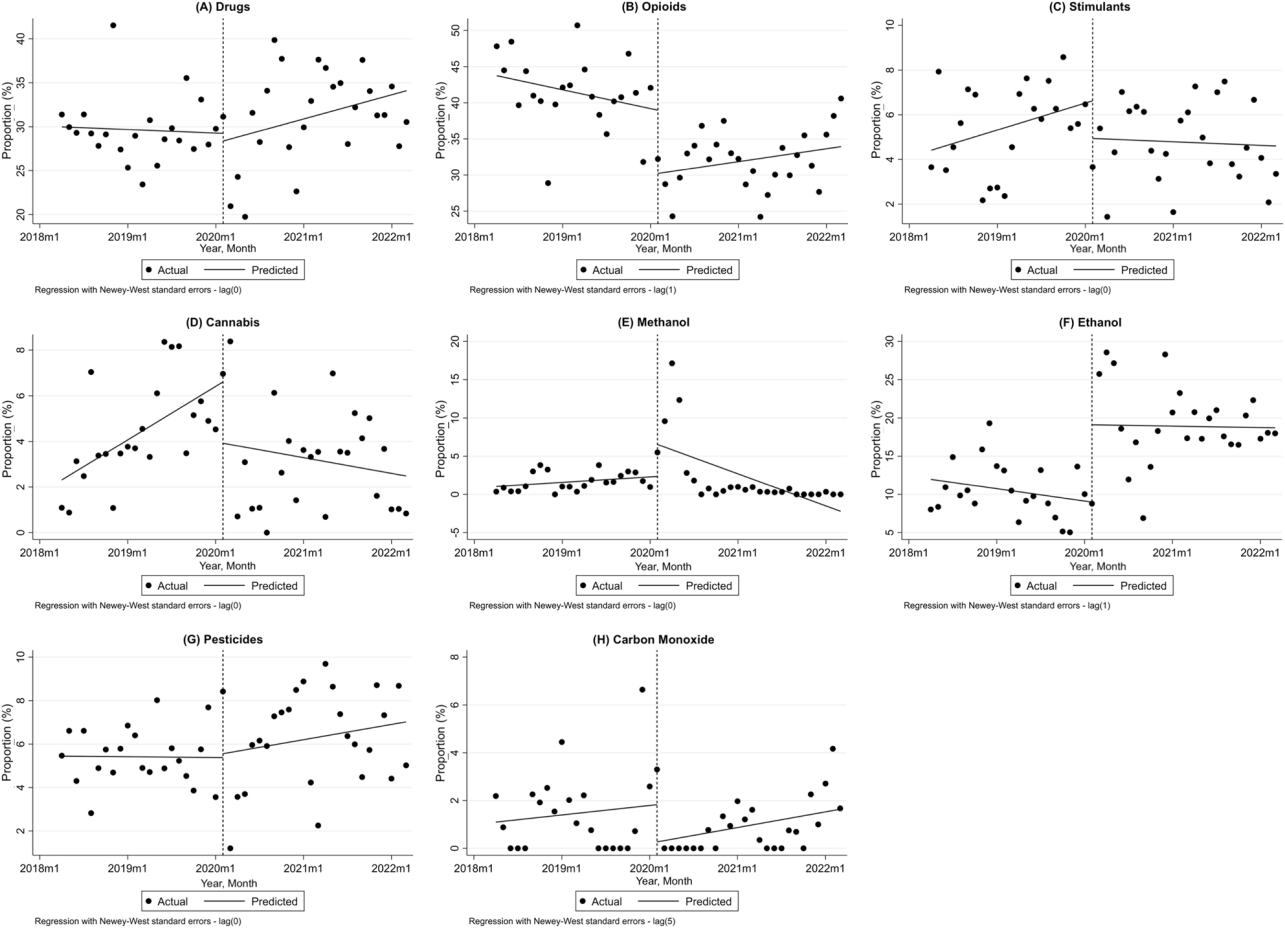
Interrupted time series analysis of the proportion (%) of poisonings from each category before and after the COVID-19 pandemic; (**A**) Drugs, (**B**) Opioids, (**C**) Stimulants, (**D**) Cannabis, (**E**) Methanol, (**F**) Ethanol, (**G**) Pesticides and (**H**) Carbon Monoxide; Dashed line represents COVID-19 outbreak; *m* month.

The trends of the proportion of each type of poisoning from the overall poisoning cases are shown in Fig. 3A–H. The statistics and ITS analysis results are described in Tables 5 and 6.

**Table 5:** The interrupted time series analysis for the ratio of poisoning due to drugs, opioids, stimulants, and cannabis.

Variable	Coefficient	Standard error	p-value	95% CI
Drugs				
Constant	30.022	1.34	< 0.001	27.321 to 32.723
Slope before	− 0.034	0.087	0.698	− 0.209 to 0.141
Level change	− 0.869	2.571	0.737	− 6.051 to 4.312
Slope change	0.263	0.158	0.103	− 0.056 to 0.581
Slope after	0.229	0.132	0.09	− 0.037 to 0.494
Opioids				
Constant	43.963	2.07	< 0.001	39.792 to 48.135
Slope before	− 0.217	0.143	0.137	− 0.505 to 0.072
Level change	− 8.756	2.545	**0.001**	− 13.886 to − 3.628
Slope change	0.365	0.182	0.063	− 0.021 to 0.752
Slope after	0.148	0.125	0.24	− 0.103 to 0.4
Stimulants				
Constant	4.313	0.867	< 0.001	2.565 to 6.061
Slope before	0.1	0.055	0.073	− 0.01 to 0.211
Level change	− 1.686	0.912	0.071	− 3.526 to 0.153
Slope change	− 0.114	0.072	0.121	− 0.259 to 0.031
Slope after	− 0.013	0.047	0.776	− 0.108 to 0.081
Cannabis				
Constant	2.116	0.746	0.007	0.613 to 3.619
Slope before	0.195	0.059	**0.002**	0.0766 to 0.314
Level change	− 2.685	1.374	0.057	− 5.454 to 0.084
Slope change	− 0.253	0.089	**0.007**	− 0.431 to − 0.073
Slope after	− 0.057	0.067	0.394	− 0.192 to 0.077

Significant values are in bold.

**Table 6:** The interrupted time series analysis for the ratio of poisoning due to methanol, ethanol, pesticides, and CO.

Variable	Coefficient	Standard error	p-value	95% CI
Methanol				
Constant	0.978	0.482	0.049	0.005 to 1.95
Slope before	0.06	0.032	0.071	− 0.005 to 0.125
Level change	4.187	2.03	**0.045**	0.09 to 8.274
Slope change	− 0.41	0.116	**0.001**	− 0.643 to − 0.176
Slope after	− 0.35	0.111	**0.003**	− 0.574 to 0.125
Ethanol				
Constant	12.102	1.776	< 0.001	8.523 to 15.681
Slope before	− 0.135	0.126	0.291	− 0.39 to 0.12
Level change	10.119	3.635	**0.008**	2.793 to 17.444
Slope change	0.119	0.211	0.576	− 0.307 to 0.545
Slope after	− 0.016	0.172	0.927	− 0.363 to 0.331
Pesticides				
Constant	5.447	0.577	< 0.001	4.284 to 6.609
Slope before	− 0.003	0.048	0.954	− 0.100 to 0.094
Level change	0.176	1.162	0.881	− 2.166 to 2.517
Slope change	0.061	0.078	0.434	− 0.095 to 0.218
Slope after	0.058	0.061	0.341	− 0.064 to 0.181
CO				
Constant	1.063	0.598	0.082	− 0.142 to 2.268
Slope before	0.033	0.061	0.591	− 0.09 to 0.156
Level change	− 1.556	0.92	0.098	− 3.41 to 0.298
Slope change	0.021	0.081	0.793	− 0.143 to 0.186
Slope after	0.055	0.039	0.164	− 0.023 to 0.132

Significant values are in bold.

#### Drugs

The trend for drug poisoning ratio was not significant either before or after the COVID-19 era. The level change and slope change were not significant as well (p-value = 0.737 and 0.103, respectively).

#### Opioids

While there was a mild insignificant decreasing trend in opioids proportion before the pandemic, which changed insignificantly to a mild increasing trend (slope change 0.365%, 95% CI − 0.021% to 0.752%, p-value = 0.063). However, the level change at the time of the COVID-19 pandemic initiation was significant showing a sudden decrease of − 8.8% (p-value = 0.001).

#### Stimulants

Stimulants had an increasing trend which was changed to decreasing after COVID-19 initiation, however, the slope before, after, and slope change was not significant.

#### Cannabis

The cannabis poisoning percentage from the total poisonings was rising before February 2020 (0.195%, 95% CI 0.077% to 0.314%, p-value = 0.002). There was a significant decrease in trend slope after the pandemic (− 0.253%, 95% CI − 0.431% to − 0.073%, p-value = 0.007).

#### Methanol

The methanol poisoning ratio had an increasing trend before the pandemic which was not significant. At the time of COVID-19 initiation, a sudden increase of 4.2% was observed in methanol poisoning percentage (p-value = 0.045). Moreover, the slope decreased by − 0.41% (p-value = 0.001), resulting in a − 0.35% post-COVID trend.

#### Ethanol

The slopes before and after the pandemic in addition to slope change were not significant. Only the level change in February 2020 was significant with an increase of 10.1% (p-value = 0.008).

#### Pesticides

No significant trend was observed for pesticide poisoning percentage trend before and after the pandemic. Also, the level change and slope change were not significant (p-value = 0.434 and 0.881, respectively).

#### CO

The proportion of CO poisoning was not changed in terms of trends before and after the COVID-19 pandemic. There was a mild increasing trend both before and after the pandemic.

## Discussion

To the best of our knowledge, this study was the first to investigate the poisoning patterns related to different drugs/substances before and after the COVID-19 pandemic. With the assessment of more than 13,000 patients, we found no difference between the demographic characteristics of the patients before and during the pandemic. In overall poisoning cases, while there was an increasing trend before the pandemic, there was a sudden drop in the first month of COVID-19. However, the trend slope was rising during the COVID-19 pandemic as well. Other main findings of the current study were: (1) there was a sudden decrease in the rates of all poisonings except ethanol and methanol, (2) the overall trend was rising for all poisonings other than methanol and cannabis, and (3) although all there was a sudden decrease in all poisoning admissions at the beginning of the pandemic, the proportion of ethanol and methanol poisonings had significant increases.

The COVID-19 pandemic had a profound impact on healthcare systems all around the world. These effects included decreased hospitalization from non-COVID-19 diseases and conditions and increased in-hospital mortality^20,21^. For instance, in Spain, a decrease of 22% in non-COVID medical and 33% in surgical hospitalizations was observed during the first wave of the pandemic^22^. In another study conducted in the United States as well, a steep decline was observed in admissions other than COVID-19 during the first months of the pandemic^20^. This was observed in several other studies as well^23–26^. Similarly, we found a significant decrease in poisoning admissions at our center, mostly due to the allocation of available beds to patients with COVID-19.

The global use of alcohol has increased from 1990 to 2017 and the trend is still upward^27^. The COVID-19 pandemic increased the rate of consumption and changed the patterns of using alcohol^28^. Several surveys found higher alcohol consumption in the United States during the COVID-19 pandemic compared to 2019^29–32^. In the report National Center for Health Statistics (NCHC) published in November 2022, they found a sharp increase in alcohol-related mortality with the emergence of COVID-19^33^. Although the increase rate between 2000 and 2018 was about 7% in alcohol-related deaths, this was changed to 26% from 2019 to 2020 which can be the effect of the COVID-19 pandemic. Another study in Iran found a 13.8% increase in hospital admissions due to alcohol intoxication in the first six months of the pandemic^7^. In addition to the global trend of increasing alcohol intake and the effect of the COVID-19 pandemic on this trend, Iranians believed that drinking ethanol can prevent or treat COVID-19^34^. Similar to the mentioned studies, we found a significant increase in the slope of ethanol intoxication in our center which changed the downward trend of ethanol intoxication before the pandemic to an upward trend.

Consuming and marketing ethanol in every form (e.g., beer, wine, etc.) is illegal in Iran. However, a meta-analysis found a 12% prevalence of last 1-year alcohol consumption in the general population in Iran which was 15% higher in young people^35^. This prevalence is not consistent in different regions of Iran due to cultural differences. Due to the illegal use of handmade alcoholic beverages in Iran, methanol intoxication is a public health issue. In a report, 768 intoxications were reported in 23 days, of which 69 died. Our study showed that the trend was upward for methanol intoxication before the pandemic. The COVID-19 emergence increased methanol intoxication in our center which can be explained by several reasons including the shortage of ethanol at the beginning of the pandemic as ethanol was mainly used for hand sanitizers in addition to the myth that ethanol can prevent and treat COVID-19 which increased the use of handmade beverages in Iran resulting in more methanol intoxication.

The Centers for Disease Control and Prevention (CDC) reported that about 50% of deaths due to drug overdose in the United States involved synthetic opioids^36^. This study found that the trend is upward with an increase of 1040% in opioid-related mortality from 2013 to 2019. Another study conducted by Olfson et al. found an increase in the rate of opioid overdose deaths from 2000 to 2017 in the United States; however, the proportion of opioid-related suicide reduced from 9 to 4%^37^. Although there are several challenges in collecting accurate data for opioid overdose in Iran^38^, a study estimated that about 65% of drug-related deaths in Iran are due to opioid overdose^39^. Similar to the global trend, we found an upward trend for opioid overdose in our center. Interestingly, the pandemic dramatically increased the slope of opioid overdose. This finding is in line with a study conducted in the United States which showed that COVID-19 significantly increased the rate of opioid-related deaths^8^.

Regarding total drug poisonings, rising trends were observed both before and during the pandemic. Drugs investigated comprised benzodiazepines, anti-depressants, anti-psychotics, and analgesics. The change in poisoning trends from these drugs might reflect the effect of the pandemic on the mental status of populations, attributable to social distancing and isolation, economic problems, and uncertainty about the future of the pandemic^40–42^. Several early studies reported conflicting results for benzodiazepine use after the initiation of the COVID-19 pandemic, with some reporting an increase in their use^43,44^, and some reporting a decrease^45,46^. A more recent study conducted in Spain which compared the patterns of benzodiazepine use and misuse in the first two years of the pandemic and compared it with the previous 2 years found that benzodiazepines used were significantly higher during the COVID-19 time, in particular clonazepam, the most frequent benzodiazepine consumed^47^. In anti-depressant use, an ITS study conducted in Israel, the use of anti-depressants changed from a decreasing trend to an increasing trend during the pandemic^48^. This was observed in nationwide data from Europe and the United States as well^49,50^. This further emphasizes the substantial risk that the pandemic posed to people’s mental health to the extent that it is described as a “mass social trauma”^51^. All these increased uses of drugs increase the chance of abuse and poisoning from them.

Stimulant poisoning had an increasing trend before the pandemic, however, there was a sudden decrease at the beginning of the pandemic. Stimulants consisted of amphetamines and cocaine. In a study conducted on young children, no immediate change was observed in cocaine and amphetamine ingestion rates at the pandemic initiation time^10^. However, a decreasing trend was observed for amphetamine ingestion after the pandemic. Similar to our study’s findings, in an interesting study that assessed the use, price, and availability of several drugs and substances worldwide, a decrease in amphetamine and cocaine use was observed during the pandemic^52^.

As the most widely used illicit substance worldwide, cannabis was estimated to have 192 million users in 2018^53^. Although opium has long been the main illicit drug used in Iran, in recent years, cannabis has also increased use mostly among high school and university students^53,54^. This increasing trend was also reflected in poisoning patterns found in our study. However, the pandemic led to a sudden decrease in the poisoning related to cannabis and also the trend. This is in line with findings from a community survey in the United States^55^. Conversely, in the global estimates, 42% of countries reported increased use of cannabis^52^. It should be noted that there might be increased use of marijuana during the pandemic due to the mental effects of the pandemic, however, the poisoning which stems from the dangerous use of cannabis might have decreased.

Finally, there is not much reported data regarding poisoning from pesticides in the literature. However, an increasing trend after the pandemic has several implications for policymaking. Intentional and unintentional uses should be addressed as the pesticide’s use has increased in recent years^56^.

Our study has several policy implications which should be highlighted. First, is the fact that there was a sudden decrease in the rate of poisoning while the trend was increasing for most of the substances. Policymakers should take this into consideration in similar situations such as pandemics or even endemics. The increase in methanol and ethanol intoxication is of high importance since it can be a potentially lethal condition with similar events in the past in the country. Educating the normal population and preparing for this rise among healthcare systems in these conditions could be doable actions for future crises. Future studies should focus on the impact of other aspects of the COVID-19 pandemic such as vaccination and each wave of the COVID-19 pandemic.

Despite investigating a large number of patients for assessment of trends in a referral center of poisoning in Iran, this study has some limitations. First, due to the single-center nature of our study, the generalizability of our findings might be threatened. Second, there are some inherent biases for ITS analysis, including history bias, cofounders, and the impact of other interventions and policies during the assessed period^57,58^. Third, although one of our findings might be a sudden reduction in poisoning-related hospitalizations, the fear of referring to hospitals and social distancing might have been effective. Finally, several factors such as the introduction of vaccines, different waves of COVID-19, and lockdown policies might be other determinants of drug/substance abuse that might not be interpretable with a single stratification into pre-COVID and COVID time. All of these suggest further research on these trends considering these factors and comparing the effect of each on overall poisonings.

## Conclusion

Although the COVID-19 pandemic has been announced to be not a major challenge anymore, there are several lessons that can help policymakers in the future. The impact of a pandemic on the trends of poisoning is one of the most affected ones. Our findings suggest that although there was a sudden fall in all poisonings at the beginning of the COVID-19 pandemic, the trend slope increased. Among poisoning agents, ethanol and methanol had immediate increases at the initiation of the pandemic which was accompanied by a decreasing trend during the pandemic for methanol. Opioids, drugs, stimulants, and ethanol had rising trends during the COVID-19 pandemic. Public health policies should benefit from these results for designing and making preventive plans for future situations^59^.

## Data Availability

The datasets used and/or analyzed during the current study are available from the corresponding author upon reasonable request.

## Abbreviations

COPD: Chronic obstructive pulmonary disease
EHR: Electronic health records
ICD: International classification of diseases
ITS: Interrupted time series
SARS-CoV-2: Severe acute respiratory syndrome coronavirus 2
SD: Standard deviation
